# Statistical Analysis of Protein-Ligand Interaction Patterns in Nuclear Receptor RORγ

**DOI:** 10.3389/fmolb.2022.904445

**Published:** 2022-06-15

**Authors:** Bill Pham, Ziju Cheng, Daniel Lopez, Richard J. Lindsay, David Foutch, Rily T. Majors, Tongye Shen

**Affiliations:** ^1^ Department of Biochemistry and Cellular and Molecular Biology, University of Tennessee, Knoxville, TN, United States; ^2^ UT-ORNL Graduate School of Genome Science and Technology, Knoxville, TN, United States

**Keywords:** protein-ligand interaction, statistical analysis, nuclear receptor, constitutive activity, inverse agonist

## Abstract

The receptor RORγ belongs to the nuclear receptor superfamily that senses small signaling molecules and regulates at the gene transcription level. Since RORγ has a high basal activity and plays an important role in immune responses, inhibitors targeting this receptor have been a focus for many studies. The receptor-ligand interaction is complex, and often subtle differences in ligand structure can determine its role as an inverse agonist or an agonist. We examined more than 130 existing RORγ crystal structures that have the same receptor complexed with different ligands. We reported the features of receptor-ligand interaction patterns and the differences between agonist and inverse agonist binding. Specific changes in the contact interaction map are identified to distinguish active and inactive conformations. Further statistical analysis of the contact interaction patterns using principal component analysis reveals a dominant mode which separates allosteric binding vs. canonical binding and a second mode which may indicate active vs. inactive structures. We also studied the nature of constitutive activity by performing a 100-ns computer simulation of apo RORγ. Using constitutively active nuclear receptor CAR as a comparison, we identified a group of conserved contacts that have similar contact strength between the two receptors. These conserved contact interactions, especially a couple key contacts in H11–H12 interaction, can be considered essential to the constitutive activity of RORγ. These protein-ligand and internal protein contact interactions can be useful in the development of new drugs that direct receptor activity.

## Introduction

The nuclear receptor (NR) superfamily is a group of important transcription factors that detect the presence of specific compounds using their ligand-binding domain (LBD) and respond by modulating gene transcription, which is directed through interaction between specific DNA response elements and the DNA-binding domain (DBD) and interaction between co-activators and the LBD (Helsen et al., 2012; Weikum et al., 2018). Well-known examples of the NR superfamily include estrogen receptor, androgen receptor, glucocorticoid receptor, vitamin D receptor, peroxisome proliferator-activated receptor, retinoid receptor, thyroid hormone receptors, and many others. While the structures of the DBD and the LBD are highly conserved, there is a highly varied and largely unstructured N-terminal domain (NTD) that also plays an important role in the function of these transcription factors (Kumar and Thompson, 2003; Simons et al., 2014).

One of the important NRs is called RAR-related orphan receptor (ROR), since initially ROR was discovered as an orphan receptor that is related to retinoid acid receptor (RAR) (Solt and Burris, 2012; Zhang et al., 2015). ROR was found to play important roles in regulating immune responses and circadian rhythm (Takeda et al., 2012; Cook et al., 2015). Furthermore, ROR was also found to be one of the few NRs that are constitutively active, meaning the receptor exhibits high basal activity (active without ligand). Since a hyperactive ROR can be tied to autoimmune diseases such as multiple sclerosis and rheumatoid arthritis, identifying potent inverse agonists to regulate ROR is of interest (Zhang et al., 2015). One essential question arises as to how one can efficiently obtain details of the protein-ligand interaction and predict how ligands affect the protein conformation, i.e., turn on or off ROR activity. As detailed below, this remains a puzzle as the ligand-protein pairing for NRs is highly sensitive.

**FIGURE 1:**
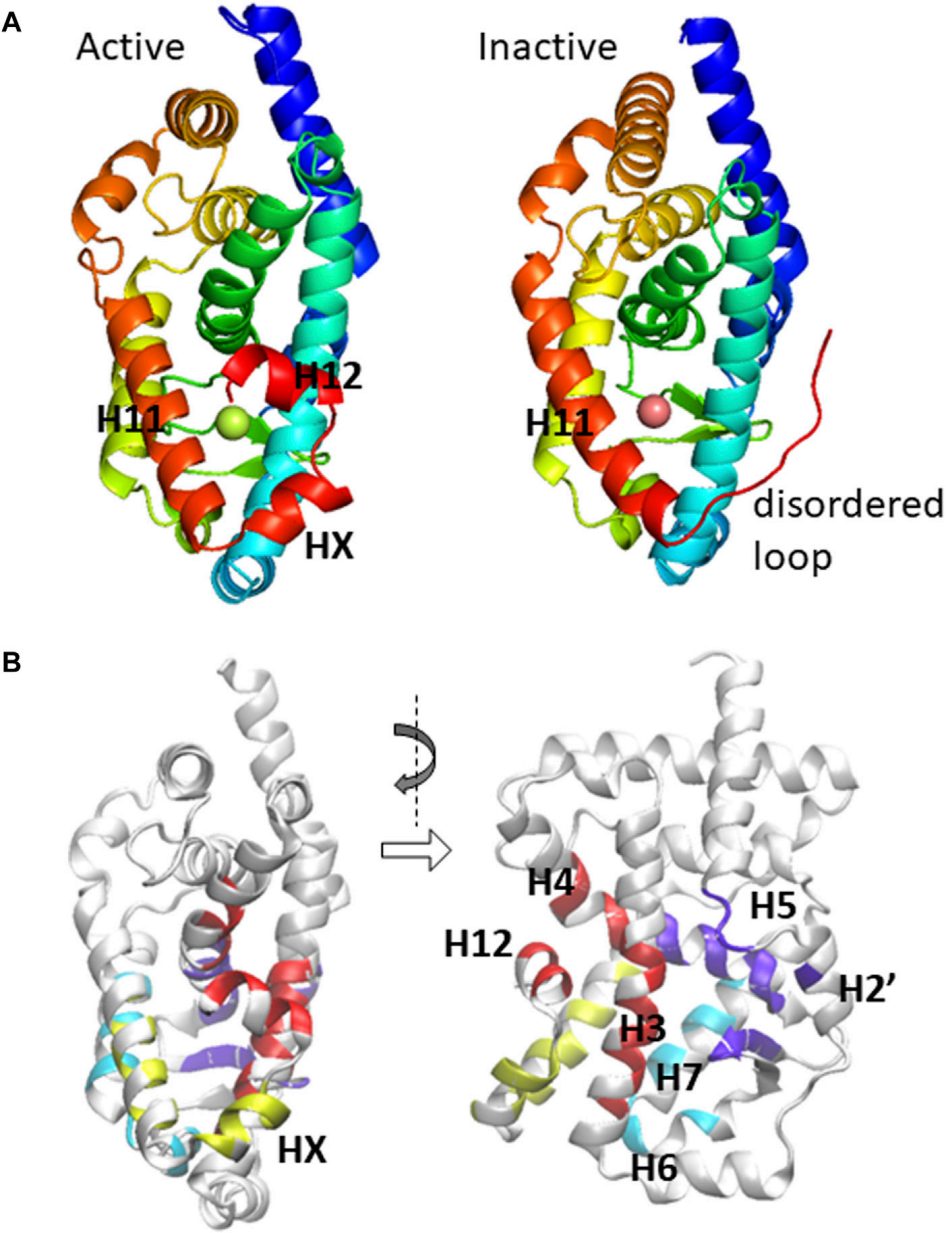
**(A)** The ligand-binding domain of RORγ is displayed in active (PDB: 3L0L) and inactive (PDB: 4QM0) conformations. The important secondary structural elements that undergo conformational change are located at the C-terminal region and are explicitly labeled. **(B)** The residues that potentially form direct contact with ligands are shown in a canonical front view and a side view.

Various structural biology and chemical biology studies have focused on ROR-ligand interactions. Among the three subtypes of ROR (RORα, RORβ, and RORγ), RORγ appears to be very important with the most structural data available, and thus we focus on examining the LBD of the γ-subtype in this study. Indeed, there have been more than 100 X-ray crystallography structures reported in the Protein Data Bank (PDB), all of which are in the monomer form having the identical protein sequence while the only differences are the unique identity of ligand(s) that forms a complex with RORγ. In many previous reports, a set of similar ligands was used to probe the cellular activity and/or biophysical properties of ROR induced by ligand binding. Similar to other NRs, binding of an agonist to the LBD leads to a conformational change that facilitates a more favorable interaction with the co-activators at the activation function 2 (AF2) region of the LBD (Weikum et al., 2018). Alternatively, when an inverse agonist binds to the LBD, co-activator binding becomes inhibited due to (at least in part) the structural changes in helices H10, H11, and/or H12 (Li et al., 2017; Noguchi et al., 2017; Gong et al., 2018). Two mechanisms of inverse agonism that have been observed include: 1) a disorder of helix H12, which would otherwise form part of the binding pocket, reduces available agonist interaction sites and 2) the formation of a “kink” between helices H10 and H11 consequently obstructs the co-activator binding site formed by helix H12, as shown in Figure 1.

Often, researchers found that whether specific ligands can turn ROR on or off is quite sensitive. For example, several studies showed that a slight modification of a known agonist or inverse agonist can switch its properties. Specifically, there were reported pairs of ligands (obtained from tertiary sulfonamides, biaryl amides, tertiary amines, benzoxazinones, and other families) that bind at the same binding site; however, the shorter of the two is an agonist while the longer ligand is an inverse agonist or vice versa, exemplified by PDB pairs: 4WPF/4WQP, 5IZ0/5IXK, 6NWU/6NWS, and 6R7K/6R7J (agonist/inverse agonist-bound structures) (Yang et al., 2014; René et al., 2015; Wang et al., 2015a; Marcotte et al., 2016; Gong et al., 2018; Wang et al., 2018; Strutzenberg et al., 2019; von Berg et al., 2019). Using 5IZ0/5IXK as an example, M358 was reported to interact with an inverse agonist ligand Bio399 (synthetic benzoxazinone) and consequently, it affects residue F506 and changes the protein conformation into an inactive form (PDB: 5IXK). In contrast, a similar ligand Bio592 has nearly identical contact interactions with the rest of the binding pocket while lacking the contact with M358, which in turn keeps RORγ in an active conformation (PDB: 5IZ0) (Marcotte et al., 2016). Meanwhile, another intriguing study found that lengthening or shortening modifications of a specific agonist (biphenyl-ethylsulfonyl-phenyl-acetamide) leads to inverse agonism (Wang et al., 2018). As different studies reported different local trigger spots for inverse agonists, one may want to consolidate ROR-ligand interactions and rethink the canonical view that the ligand-directed action comes from a fixed chemical group of the compound with a specific residue of the binding pocket. Instead, the ligands examined in these studies are diverse and distinct from one study to another. It appears that a specific chemical group is not enough to determine the effect of a molecule, and yet the ligand identity clearly affects the interactions with the binding pocket and subsequently the protein activities. For nuclear receptors, there is a challenge on how to connect the ligand identity with directed structural changes and subsequent activities.

We think that a statistical analysis of extensive structural information where one collectively examines protein-ligand contact interaction patterns may provide insight into this challenge. For this work, we studied 136 RORγ structures: 132 with one ligand (or ligand fragments) bound (100+ distinct ligands) from X-ray crystallography experiments (Jin et al., 2010; Fujita-Sato et al., 2011; Fauber et al., 2013; Fauber et al., 2014; van Niel et al., 2014; Yang et al., 2014; Chao et al., 2015; Muegge et al., 2015; René et al., 2015; Santori et al., 2015; Scheepstra et al., 2015; Wang et al., 2015b; Wang et al., 2015c; Enyedy et al., 2016; Hirata et al., 2016; Hintermann et al., 2016; Marcotte et al., 2016; Olsson et al., 2016; Ouvry et al., 2016; Xue et al., 2016; Kallen et al., 2017; Kummer et al., 2017; Li et al., 2017; Noguchi et al., 2017; Carcache et al., 2018; Fukase et al., 2018; Gege et al., 2018; Gong et al., 2018; Kono et al., 2018; Narjes et al., 2018; Noguchi et al., 2018; Sasaki et al., 2018; Schnute et al., 2018; Shirai et al., 2018; Wang et al., 2018; Amaudrut et al., 2019; Duan et al., 2019; Hoegenauer et al., 2019; Kotoku et al., 2019; Lu et al., 2019; Marcoux et al., 2019; Sato et al., 2019; Strutzenberg et al., 2019; Tanis et al., 2019; von Berg et al., 2019; Yukawa et al., 2019; Zhang et al., 2019; Cherney et al., 2020; Duan et al., 2020; Gege et al., 2020; Harikrishnan et al., 2020; Jiang et al., 2020; Liu et al., 2020; Meijer et al., 2020; Nakajima et al., 2020; Shi et al., 2020; Vries et al., 2020; Zhang et al., 2020; Lugar et al., 2021; Meijer et al., 2021; Nakajima et al., 2021; Ruan et al., 2021; Yang et al., 2021). Additionally, there was also a report of 12 structures (with two ligands bound) (de Vries et al., 2021). The full list of PDBs and their associated properties can be found in the Supplementary Data File S1. We did not include the double-liganded structures in the analysis since almost all the ligands in those structures have been crystalized with RORγ previously, and thus these ligand-protein contacts have already been included in our analysis. There might be new RORγ structures deposited in the PDB since the time of our structural bioinformatics research, and any newly reported RORγ structures after that time would not be included in the current analysis. However, we expect that the results of our statistical analyses and the conclusions drawn should still hold.

The current work has two main focuses. The first one is the statistical analyses of the protein-ligand interactions, obtained from previous experimental studies in which each examined ligand or multiple ligands interact with the binding pocket. The comparison across all ligands will provide a more comprehensive picture of the molecular interactions that differentiate between agonists and inverse agonists, and potentially illustrate the mechanism (structural change) by which each ligand imposes its effect. The second focus is the nature of NR constitutive activity. The RORs have a high basal activity and thus they are considered to be active without any ligands. Such constitutive activity of receptors, including many prominent examples from the NR and GPCR families, are difficult to study experimentally at times. Often, receptors, including RORγ, do not have structures resolved experimentally in the absence of ligand. Computational study of an apo conformation may provide clues on how they function (Pham et al., 2019a; Rosenberg et al., 2019). Within the nuclear receptor superfamily, only a few wild-type receptors display constitutive activity. Constitutive androstane receptor (CAR) is also deemed to be constitutively active as suggested by its name (Dussault et al., 2002; Xu et al., 2004). The CAR protein functions as a xenobiotic sensor, which detects foreign substances such as drug molecules and metabolizes them primarily in the liver (Xie et al., 2003; Wang et al., 2012). Additionally, a couple other NRs were also suggested to have a high basal activity, such as ERR and SF-1/LRH-1 (Schimmer and White, 2010; Huss et al., 2015). Often, they have a relatively small binding pocket. A previous computational study performed on CAR has shown some essential protein contacts contributing to the constitutive stability of the unliganded CAR (Pham et al., 2019a). By comparing CAR with RORγ, we may gain insights into the important protein interactions that help facilitate the constitutive activity of nuclear receptors in general.

## Methods and Systems

### Crystal Structure Ensemble of RORs With Various Ligands Bound

The statistical analysis includes a total of 136 X-ray crystal structures. Only four of the structures (PDB: 5K38, 5VB3, 5X8U, and 5X8W) are absent of a ligand and the other 132 structures contain a single ligand at the ligand-binding pocket. The binding pocket mentioned refers to either a canonical, largely enclosed ligand-binding pocket or an adjacent, more exposed allosteric binding site. We did not include the 12 structures with double ligands (one each at canonical and allosteric binding sites).

For the protein component of the complex, all 136 structures contain a single chain of LBD of RORγ. Note that we also include RORγt, an isoform of RORγ that is selectively expressed in the thymus. Although the sequence of RORγt is 21-residues shorter than RORγ at the N-terminal domain (NTD) due to alternative splicing, both RORγt and RORγ have an identical LBD. Among 136 structures, only a few of them (PDBs: 4NB6, 6O98, 6XFV, and 7JH2) were reported using RORγt indices for their residues while the rest used RORγ. Six of them are from gibbon ROR, which only contains a double substitution (K469A/R473A) from human UNP P51449. Another 34 PDBs are single point mutants at C455 (mostly C455S, occasionally C–H or C–E mutations were reported). By visual inspection, these mutations or substitutions are far from the ligand binding pocket, e.g., C455 is at helix H9, thus none of them are directly involved with the protein-ligand contact interaction. Therefore, we do not treat them separately from the wild-type RORγt.

For the 132 structures containing only one ligand, there are a total of 125 distinct ligands. Notably, four PDB pairs (4YPQ and 5C4O, 5K3M and 5X8S, 5NI5 and 5NU1, 5APJ and 5APK) share the same ligands and four additional PDBs (4NB6, 5EJV, 5K3L, and 5NTQ) all share the same synthetic ligand T0901317 (also an agonist for LXR). Even though these pairs and groups may share the same ligand, the protein conformations are not necessarily the same. For example, despite sharing the same inverse agonist, 5APJ is active (due to a fused coactivator) while 5APK is in an inactive conformation (Olsson et al., 2016). The structures of 4YPQ and 5C4O are in different space groups (Scheepstra et al., 2015), whereas 5NI5 and 5NU1 are bound to different coactivators. A unique case, 5G44, contains three ligand fragments in the binding pocket as it was obtained from a cosolvent engineering study (Xue et al., 2016). We treated this three-ligand “cocktail” as a single ligand. One of the 116 PDBs, 6W9J, was removed from the structure database and replaced by 6XAE after we started our study. Since 6W9J has an identical ligand as the one from another structure already in this database, 6W9H, we have included 6XAE in the below analysis and excluded 6W9J. As mentioned in the Introduction section, a few additional structures of ROR-ligand complexes were reported after our search but we did not include them in the study.

### Structural Ensemble of Apo ROR From MD Simulation

To construct the initial conformation of the unliganded ROR system, we used a crystal structure of a coactivator-fused ROR (PDB: 5VB3) that is absent of any ligands (Li et al., 2017). The structure is deemed to be in an active conformation, and it was selected from a set of four apo crystal structures (PDB: 5K38, 5VB3, 5X8U, and 5X8W) which are void of agonist ligand binding (Li et al., 2017; Noguchi et al., 2017). It is worth noting that three structures (PDB: 5VB3, 5X8U, and 5X8W) within that set are not fully unliganded since they are either bound to or fused with the coactivator peptide (CoA), while the other structure (PDB: 5K38) without CoA binding has an incomplete C-terminus. As a fused protein complex of the ligand-binding domain of ROR and CoA, the CoA component is believed to assist ROR in remaining in the active conformation. Interestingly, the CoA effect is so strong that the inverse agonist-bound form of this fused protein is still in the active conformation as the PDB 4YMQ shows (Muegge et al., 2015). To simulate our fully apo system, we removed the fused CoA segment from the ROR protein of the crystal structure. The protein contains 243 residues with internal indices (1–243) corresponding to the standard RORγ (UniProt: P51449) A265–S507.

The AMBER14SB forcefield was used for the protein molecules of the simulation, whereas the TIP3P model was used to solvate the system with 11,622 water molecules in a rectangular box. The protonation status of the residues was determined by H++ (Anandakrishnan et al., 2012) at pH of 7.0 and assigned accordingly: Asp and Glu are deprotonated, Arg and Lys are protonated, and all His are singly protonated at the *ε* position, except His452 and His479 which are protonated at the *δ* position. Two Cl^−^ counterions were added to neutralize the system.

After the initial setup of the system, we conducted minimization, heating, and production runs using NAMD. NPT simulations were used for the system with T = 300 K and *p* = 1 atm. The production run time was 100 ns after an initial 5 ns equilibration. The time step was 2 fs, and snapshots were collected every 1 ps.

### Statistical Analysis

Contact matrices are calculated to render the structure information at residue-residue contact resolution (Johnson et al., 2015; Clark et al., 2016; Johnson et al., 2018). For the calculation of residue-residue and residue-ligand contacts of these 136 PDBs, hydrogen atoms are excluded from all but the 10 ligands for which they were explicitly reported. Since the hydrogen atoms of proteins were not explicitly reported either, we remove hydrogens from all of the PDBs to obtain a uniform resolution (heavy atom only) of the protein complex systems. A contact $a_{ij}\;$ between two components,  i and  j (a pair of amino acid residues or a residue and a ligand), is considered formed $a_{ij}=1$ if the minimum distance between heavy atoms from the two components is within 4.5 Å, otherwise $a_{ij}=0$ . For the corresponding processing of simulation results, the distance cutoff is 4.2 Å using an all-atom resolution (hydrogen included).

Several analysis methods are used to further render the information contained in the contact matrix ensemble. Besides the principal component analysis (PCA) (Jolliffe, 2002; Brunton and Kutz, 2019) of contacts, statistical analyses such as hierarchical clustering and construction of a dendrogram can be used to classify the protein structures (contact maps), the residues of binding pocket, and the ligands. The distance score between two protein contact matrices $a_{ij}$ and $b_{ij}$ is defined as $N_{10}=\underset{ij}{\sum }{\left(a_{ij}–b_{ij}\right)}^{2}$ . An alternative definition based on $1/\underset{ij}{\sum }\left(a_{ij}\cdot b_{ij}\right)=1/N_{11}$ provides similar clustering results. Here, $N_{10}$ is the number of the elements that are different (i.e., logic gate XOR performed) and $N_{11}$ is the number of elements that are 1 (contact formed) in both cases. Note that sequentially neighboring contacts (those between residue i and i+1, i+2) are not counted. Since different proteins have different starting and ending positions for their contact maps, we use a common region (between 276 and 475) and thus a contact matrix size of 200 ×200 for this distance calculation.

For protein-ligand interactions, we used an I-PCA style of contact statistical analysis (Lindsay et al., 2018). The I-PCA method was initially developed to reveal internal domain structures of semi-structured biopolymers, from large-scale chromosome structures to intrinsically disordered proteins (Das et al., 2020; Lindsay et al., 2021). In those cases, each row (or column) of the mean contact map of the structure ensemble is treated as a linear set of contact variables (the number of rows is the number of monomer units of the system) that symbolize the contact interaction with other unlabeled monomers. Here, we generalize this idea to protein-ligand contacts, i.e., protein residues have a contact variable L that forms unspecified contact with ligands, i.e., $L_{i}=1$ if residue  i is in contact with the ligand or 0 otherwise. Thus, we emphasize the correlation of contact formation between residue  i with the ligand while residue j simultaneously forms contact with the ligand. The covariance matrix is $C_{ij}=<\delta L_{i}\cdot \delta L_{j}>=\overset{K}{\underset{\alpha =1}{\sum }}\delta L_{i}^{\alpha }\cdot \delta L_{j}^{\alpha }/K$ . Here, $\text{δ}L_{i}^{\text{α}}=L_{i}^{\text{α}}-<L_{i}>$ is the protein-ligand contact fluctuation of residue i and symbol the < > indicates an average performed over K=132 different protein-ligand contact patterns. The emphasis is on which residue makes contact, while the details of ligand structure are not emphasized here. One can expect that applying I-PCA may sort out the dominant contact interaction patterns between residues and ligands.

## Results and Discussion

### Conformations With Various Ligand Binding Status Expressed by Contact Matrices

**FIGURE 2:**
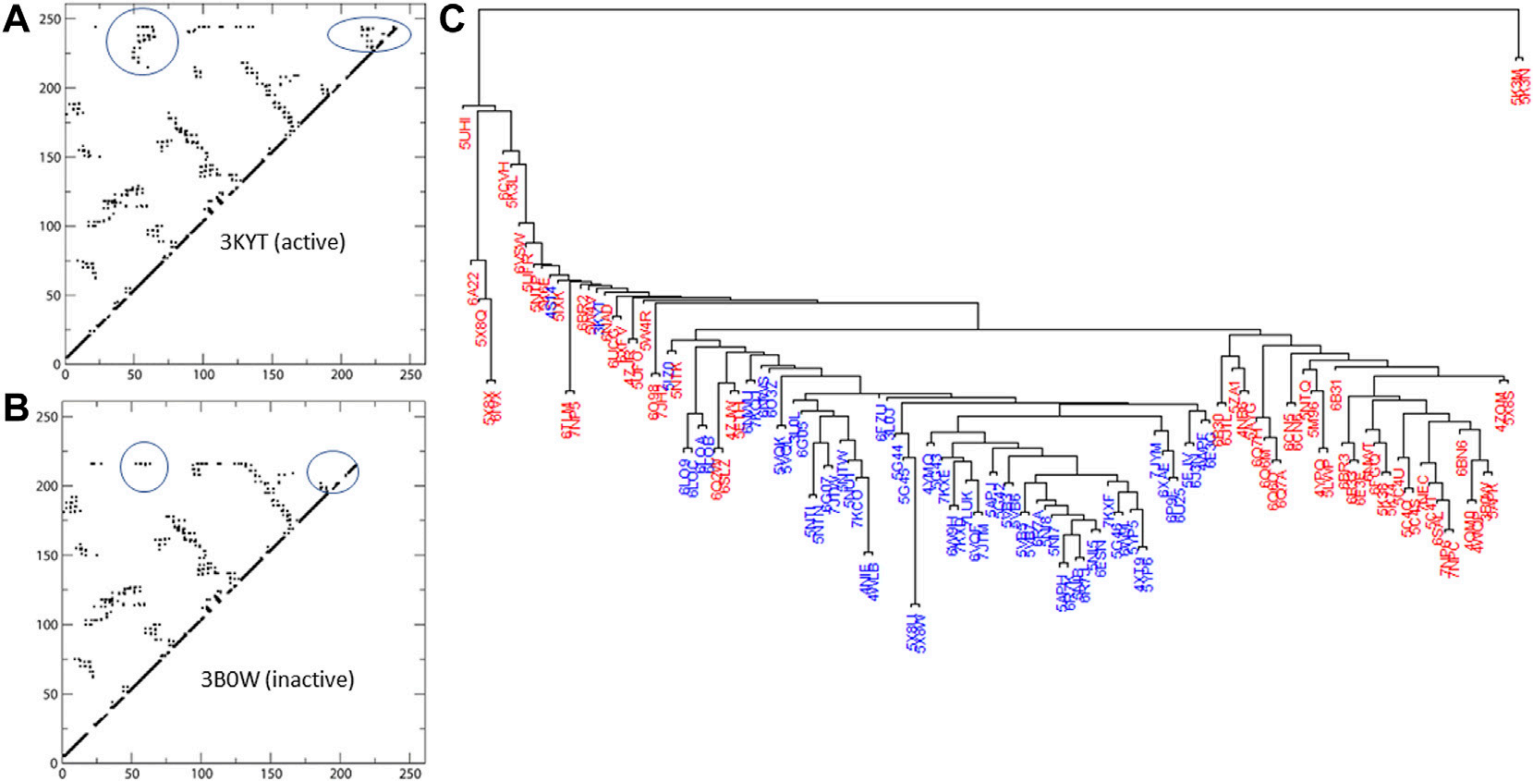
Active and inactive structures of RORγ are rendered as contact maps in **(A,B)**, respectively. Based on the similarity matrix, a dendrogram of the structures (active labeled in blue, inactive in red) at the contact level is displayed in **(C)**.

We first use contact interaction matrices to compare the conformations of RORγ structures reported in the PDB database. As mentioned in the previous section, a distance (dissimilarity score $N_{10}$ ) between two conformations measured by the similarity between the corresponding contact matrices is calculated. This contact-based similarity measurement provides a simple way of grouping similar conformations. Practically, the value of $N_{10}$ ranges from 0 to 142. The hierarchical clustering of structures characterized by these contact maps (based on the pairwise dissimilarity scores) is represented as a dendrogram in Figure 2.

With the exception of some isolated structures that branch off earlier, the bulk of the structures form two main branches in the dendrogram. As seen in Figure 2, the active structures form the majority of one branch, the active branch, whereas the inactive ones concentrate in the other branch. Note that none of the nine structures complexed with allosteric inhibitors (PDBs: 4YPQ, 5C4O, 5C4S, 5C4T, 5C4U, 5LWP, 6SAL, 6TLM, and 6UCG) is in the active branch and most of them are in the inactive branch. Two structure outliers (5K3M and 5K3N) branch off the earliest, since they have distinct conformations compared to the rest of the structures with less contacts being formed. In addition, three of the four apo structures can be found within the “active branch” of the cluster tree and the fourth is found within the “inactive branch.”

We use color labeling to demonstrate the conformation being active (blue) or inactive (red) in Figure 2. Active structures are largely located in one branch of the cluster tree. Note that our definition of active conformation is based on the features of contact matrices as stated below. We could not solely rely on the self-reported status from literature associated with the PDBs, because not all ligands were self-reported as an agonist or an inverse agonist. Additionally, the active or inactive conformation is not always linked to the ligand being reported as an agonist or an inverse agonist. In certain cases, such as a coactivator-fused ROR or due to ROR-coactivator interaction, a known inverse agonist can be “trapped” within the active conformation of the protein (e.g., PDB: 5APJ). However, by visual inspection of the contact interactions, one can clearly observe two distinct patterns of contact maps being formed. The first group of contact maps is predominantly associated with self-identified active conformations and features two regions of contacts that are absent from the second (inactive) group. One of the two regions represents contacts between H3-H4 and HX-H12, whereas the other region contains contacts between HX and H12. Examples of the active and inactive contact maps are displayed in Figure 2.

In practice, we summed the total number of contacts from two regions on the contact maps where Region one is defined as any contacts between residues i and j , i.e., (i,j) satisfy 300<i<340 and j>475 , and Region two by any contacts satisfying 470<i<495 , i<j , and j>490 . We further applied a cutoff value of 60 contacts in these two regions combined to determine whether a specific structure is active or inactive, with 60 and above considered as active. Based on this *ad hoc* cutoff criterion, we can separate all the structures into two camps of roughly equal size: 67 of 136 structures are considered active and the remaining 69 are considered inactive. This cutoff selection and the ensuing definition of structure (active vs. inactive) are also proven to be largely consistent with most self-reported or presumed classification of ligand status (agonist vs. inverse agonist). Out of these 132 ligand-bound structures, 113 have a consistent ligand identity and structure identity. There are 19 structures with a presumed or self-reported inverse agonist that yield a value slightly greater than our cutoff of 60 (mostly around 65–70), which makes them active structures by our definition. Several factors can contribute to this result. Besides the factor that a structure can be influenced by elements other than the ligand’s nature (e.g., 5APJ vs. 5APK), different experimental tests to determine the nature of the ligand being an inverse agonist or not are inconsistent. Besides, the action of the ligand binding is not a discrete value, but rather the level of effect is on a continuous spectrum.

### Ligand Binding Patterns Revealed by Statistical Analysis of Protein-Ligand Contacts

In general, the ligand binding pockets of NR can be quite large and complex. For most cases, ligands are considered to be completely enclosed inside the LBD of the receptor. A unique aspect of ROR is that it contains an allosteric binding site besides the canonical (orthosteric) binding site (Scheepstra et al., 2015). It was reported further that both sites can be occupied by ligands and exhibit a degree of communication between them (de Vries et al., 2021). Specifically, even when an agonist ligand binds to the canonical site and stabilizes the binding pocket structure, the presence of an allosteric inverse agonist can negate the agonistic effect and turn off the receptor activity (de Vries et al., 2021). Although such complex multivalent interaction is interesting to study and can have deep implications on controlling how the protein functions *via* allostery (Pham et al., 2019b), our study is limited to only single ligand-protein interaction.

A basic property of ligands which we can investigate is their size and its relationship with the ligand identity as an agonist or an inverse agonist. Here, we chose to characterize each ligand by its total number of atoms. The mean ligand size is n= 58.4 with a standard deviation of 17.5, whereas the active structures have a mean ligand size of $\;n_{a}=55.5$ and the inactive structures have $\;n_{i}=61.9$ . Although ligands in the inactive structures are slightly heavier, the difference is much smaller compared to the standard deviation of size distribution. Thus, we conclude that ligand size is not a determining factor as to whether the ligand is an agonist or not. The conclusions drawn here are insensitive to alternative definitions using molecular weight or number of heavy atoms, as these three definitions are highly correlated.

With the same noise filtering cutoff, we define the binding-pocket residues as those forming contacts with ligands in at least 10 out of 132 ligand-bound structures. As a result, we found a total of 55 residues forming the binding pocket. For comparison, a slightly more relaxed, alternative cutoff of 8 hits yields a total of 56 residues.

**FIGURE 3:**
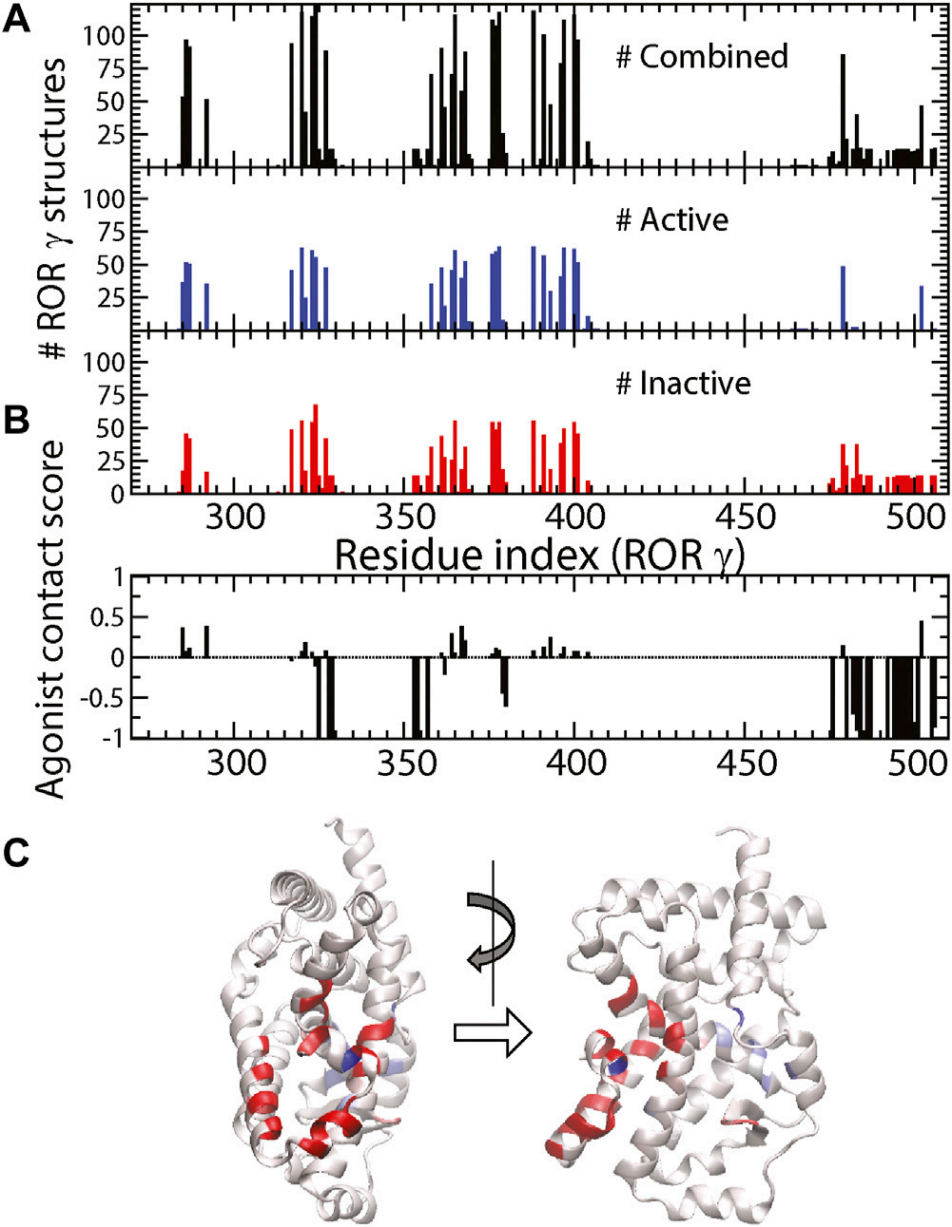
**(A)** The total number of ligand-protein contacts made by a specific residue (black), the number of ligand-protein contacts in active and inactive structures (blue and red). **(B)** The agonist contact score is displayed as a function of residue. **(C)** The corresponding values from **(B)** are color-labeled onto the 3D structure of RORγ (front and side views).

As shown in Figure 3A, the total number of ligands $N_{i}^{T}$ , is shown as a function of protein residue index i . Particularly, residues at the binding pocket form constant contacts with ligands are 320, 323–324, 365, 376–378, 388, 397, and 400 as they form contacts with a ligand in more than 80% of the structure ensemble. One can separately list the number of residue-ligand contacts $N_{i}^{T}\;$ formed in active ( $N_{i}^{A}=\sum^{\prime A}L_{i}$ ) vs. inactive ( $N_{i}^{I}=\sum^{\prime I}L_{i}$ ) conformations based on the (in)active definitions defined in the previous subsection. Here $\Sigma {’}^{A,I}$ is a restricted sum for active and inactive conformations respectively and $N_{i}^{T}=N_{i}^{A}+\;N_{i}^{I}\;$ . Furthermore, we have defined the preference of each residue as the agonist contact score, $x=\left(N^{A}-N^{I}\right)/\left(N^{A}+N^{I}\right)$ , as seen in Figure 3B. The value of x is in the range of [−1,1] , where −1 indicates residue-ligand contacts only formed in the inactive case and +1 are those only formed in the active case. Note that we filtered out residues that have minimal contacts with the ligand ( $N_{i}^{T}\leq \;9$ ) to avoid poor statistics.

It might be useful to point out that the residues with strongest negative agonist contact scores (highlighted in red in Figure 3C) are located at five specific spots (near residues 325, 328–329, 353–354, 357, and 379–380) and the C-terminus (476–506). Note that three of those spots belong to the allosteric binding pocket and the other two spots (residues 357 and 379–380) reside in the boundary between the allosteric and canonical binding pockets. These inverse agonist “hot spots” can be important for ligand design and some of them have been reported as the “trigger” for inducing inactive conformations of RORγ. For example, one of the most potent inverse agonists from isoxazole family was reported to have contact interaction with Q329, L353, and K354 (Meijer et al., 2020). Another example on the boundary is the M358 trigger (Marcotte et al., 2016) mentioned in the Introduction. Combined with molecular docking (Trott and Olson, 2010), this contact score (Figure 3B) can be further developed and applied to high throughput screening for selecting new inverse agonist ligands for RORγ. This statistical approach may also be generalized to study ligand recognition by other receptors.

**FIGURE 4:**
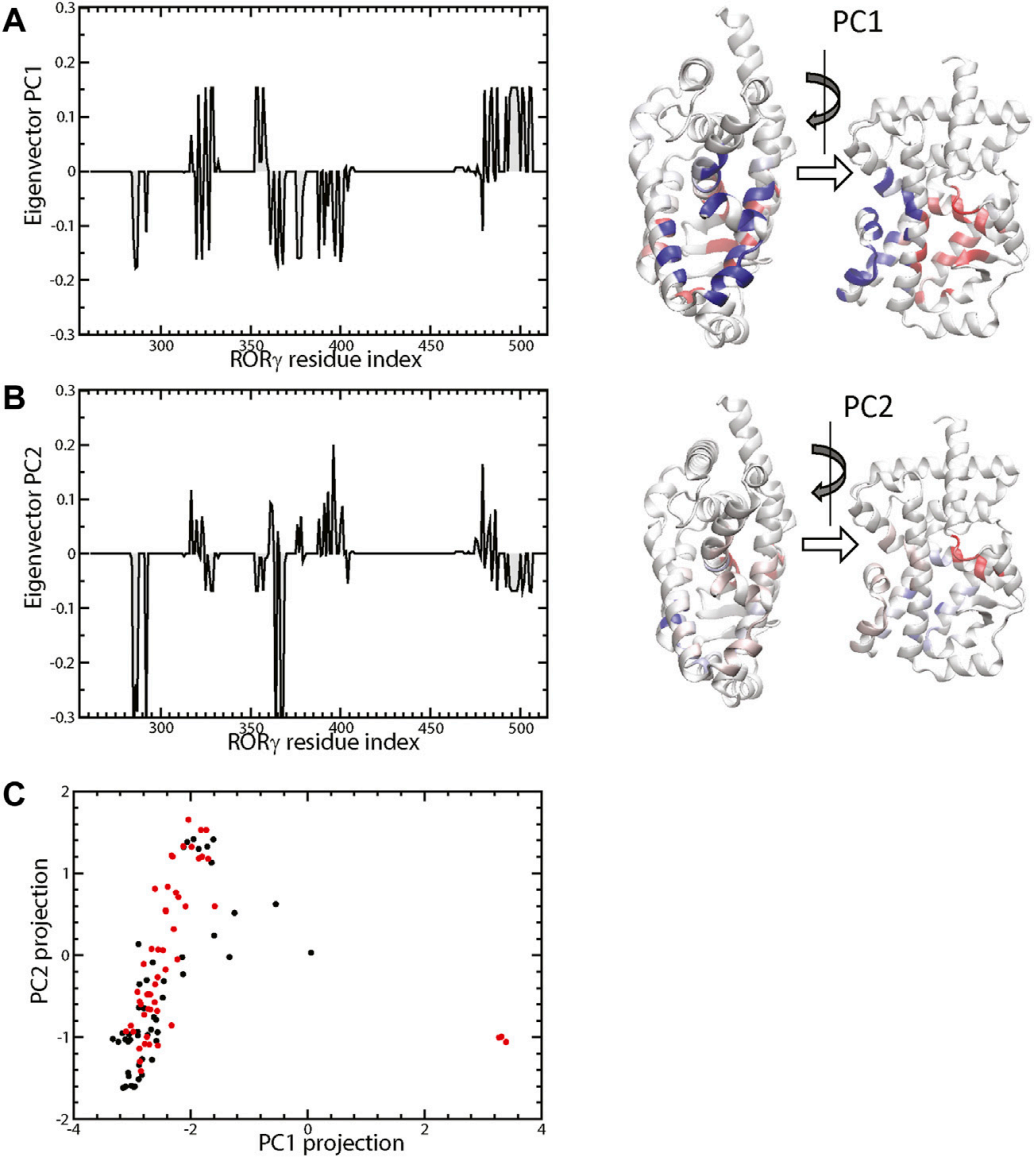
The top two eigenvectors PC1 and PC2 are shown in **(A**,**B)**, respectively. The 3D representations are colored by the elements of the corresponding eigenvectors (blue+ and red−). The projection of protein-ligand interaction from the top two PCs of the 132 structures (active labeled in black and inactive in red) is shown in **(C)**.

Besides obtaining independent statistics on the ligand contact tendency of each residue, we further investigated the concerted pattern of the residue-ligand contacts, i.e., whether residues *i* and *j* form protein-ligand contacts in sync. Various statistical analyses can be used to achieve this correlation analysis, and we use contact PCA as described in the Method section. The contact PCA on the covariance matrix of residue-ligand contacts provides the dominant patterns of residue-ligand interaction. The top eigenvectors PC1 and PC2 were presented in Figures 4A,B. We also analyzed the PC projection for PC1 and PC2, which is shown in Figure 4C. Each PC mode indicates a specific binding pattern: all residues with positive values form contacts with the ligand (i.e., not necessarily the same ligand) in sync and the same goes for residues with all negative values. Additionally, there is an anti-correlation between positive residues and negative residues. One can see that the dominant mode, PC1, largely divides residues into two groups. As ligand contacts from conformations of PDB structures (4YPQ, 5C4O, 5C4S, 5C4T, 5C4U, 5LWP, 6SAL, 6TLM, and 6UCG) mostly come from the positive group, they show up as positive PC projections, whereas the remaining conformations comprise the negative group. Overall, we found that PC1 distinguishes two binding modes: allosteric binding for the positive group and canonical binding for the negative one. The position of the allosteric binding pocket is distal to the traditional canonical binding pocket, and the ligands that interact with the allosteric binding pocket have been found to be a class of inverse agonists (Meijer et al., 2020; Vries et al., 2020; Zhang et al., 2020; de Vries et al., 2021; Meijer et al., 2021). Function-wise, these allosteric inverse agonists induce another orientation of helix H12 such that it prevents the binding of a coactivator. The second dominant interaction pattern, PC2, shows another prominent binding feature, which seems to weakly separate agonist vs. inverse agonist binding. It is interesting to point out that most conformations with an extreme positive PC2 projection are inactive conformations (red) and vice versa, an extreme negative PC2 for active conformations (black).

### Essential Contacts for Constitutive Activity Revealed by Molecular Dynamics Simulation

As mentioned in the Introduction, the high basal activity of certain NRs makes finding the mechanism of constitutive activity and inhibitors to these NRs important. Since the apo structure of NRs in general is difficult to obtain and most existing NR structures are complexed with ligand(s), we use computer simulation to sample the apo structural ensemble. Furthermore, we use statistics of residue-residue contacts to characterize the mechanism of high basal activity at the residue-residue interaction resolution.

**FIGURE 5:**
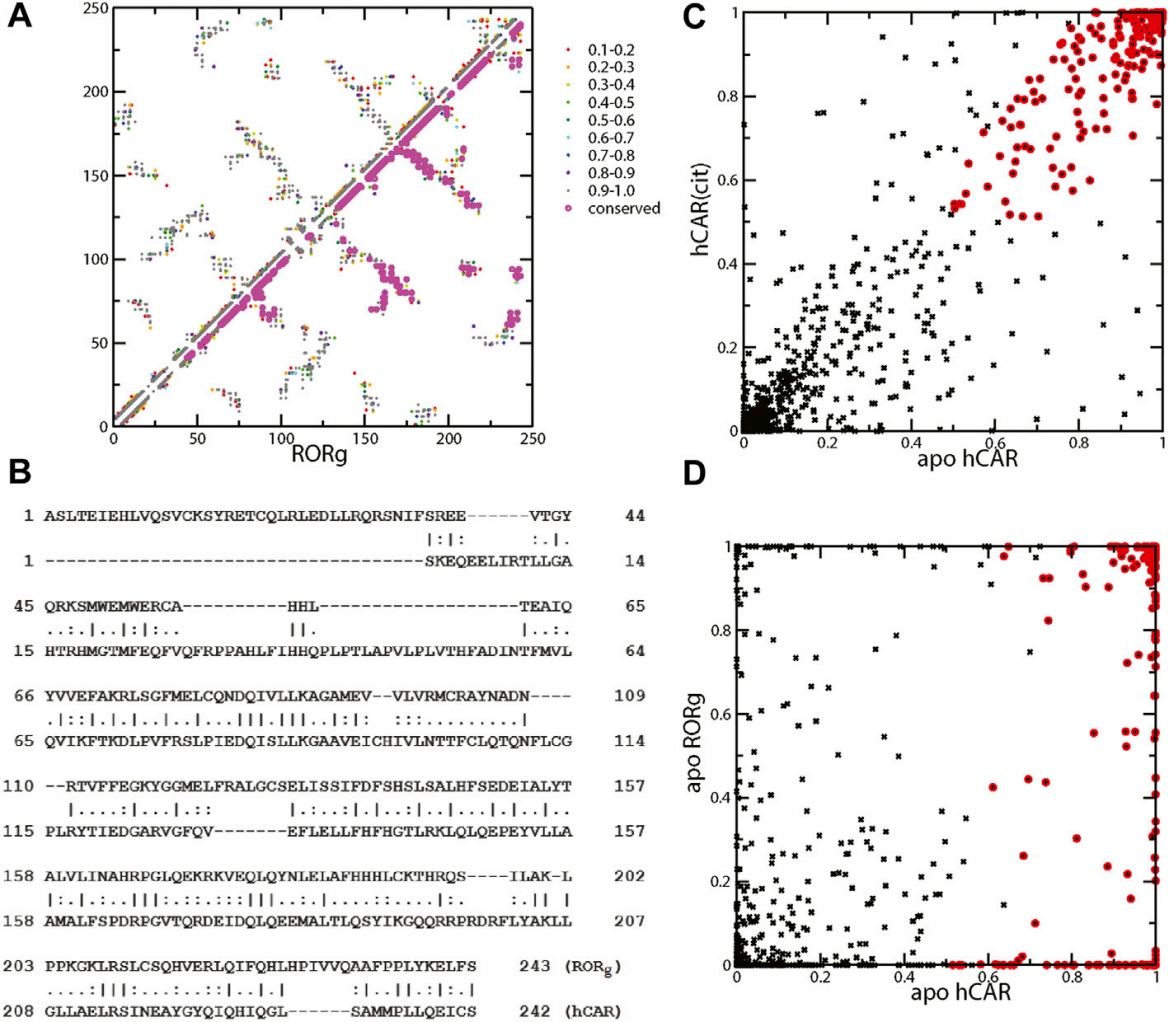
**(A)** Mean contact interaction of unliganded RORγ during simulation (upper-left triangle). The contact strength values are color-coded, and the conserved contacts are highlighted in magenta. **(B)** Sequence alignment between RORγ (UniProt: P51449) and hCAR (UniProt: Q14994) for a direct comparison between contacts. The listed index can be converted to the standard index by +264 and +106 for RORγ and hCAR, respectively, i.e., the first Ala is A265 for RORγ and the first Ser is S107 for hCAR. **(C)** The scatter plot of contact interaction between unliganded hCAR and hCAR with the agonist CITCO. The conserved contacts are labeled using red circles. **(D)** The scatter plot of contact interaction between unliganded hCAR and unliganded RORγ.

We performed a 100-ns simulation on the unliganded RORγ and analyzed the snapshots using contact analysis, which focuses on residue-residue contact interaction during the simulation. We then examined the contact interactions within the RORγ receptor and focused on identifying contacts with high interaction strength in the apo ensemble. The mean contact matrix of apo RORγ is shown in Figure 5A (upper triangle). Each element of the mean contact matrix (also termed contact frequency) is displayed on a contact map using spectrum color-labeling, ranging from rarely formed with contact ratio at 0.1–0.2 (red) to nearly always formed at 0.9–1.0 (dark gray), which largely reflects the contact interaction strength during the simulation.

There are many ways of selecting essential contact interactions that are responsible for high basal activity. Here, we focus on two aspects of conserved highly-formed contact interactions. One aspect is the conservation across different nuclear receptors and the other is the conservation between ligand-bound and apo forms. Thus, we emphasize that the essential contacts are the contacts that not only consistently show up regardless of the ligand binding status but also persist across different NRs. To address how constitutive activity can be conserved across nuclear receptors, we compare the essential contact interactions of RORγ with those of a prominent constitutively active receptor, CAR. The LBDs of both receptors are similar in size and structure. The LBD of RORγ contains 243 residues compared to 242 for CAR. Structure-wise, these two LBDs share a similar fold and both display a short helix, HX, which is unique among the LBDs of nuclear receptors. The presence of helix HX in CAR has been suggested to stabilize the active conformation of the apo form leading to the constitutive activity of CAR (Pham et al., 2019a). The sequence alignment on RORγ and CAR shows a good alignment and conserved residues in Figure 5B, especially after the first 50 residues. The sequences of the two receptors share 59 identical residues (∼20%). This alignment facilitates our comparison of residues between RORγ and CAR and the comparison of residue-residue contact interactions between the two receptors.

Before we locate the conserved contacts between NRs, we first identify the contacts that are conserved between apo and agonist ligand-bound forms. Since we only performed the apo RORγ simulation and we have previously obtained both ligand-bound and apo simulations for CAR, we use the CAR system to select the contacts between apo and agonist ligand-bound forms. Specifically, we use an *ad hoc* selection criterion of contact formation that is larger than 50% for both forms to identify the conserved contacts between apo and ligand-bound forms, and these conserved contacts are annotated with red circles as seen in Figure 5C. Furthermore, to locate the essential contacts that are conserved between different receptors, we directly compared the contact interactions of RORγ with the CAR receptor, as shown in Figure 5D. Again, the red circles are annotated for the conserved contacts selected (based on the high contact conservation between apo and ligand-bound) from Figure 5C. Finally, a subset of annotated contacts, which are the conserved contacts across NRs (defined as larger than 50% for both apo forms), is highlighted in magenta in Figure 5A (lower triangle). The conservation between CAR and RORγ contacts is quite extensive especially at the C-terminal half of the LBD, which leads to the conclusion that the mechanism of constitutive activity is similar between them. It may be of interest to investigate whether we can apply the inverse agonist ligand design of ROR to another system, e.g., to explore a potential allosteric binding site of CAR.

Based on the mean contact strength (Figure 5A) and the sequence alignment (Figure 5B), we found that the contacts between helices H11 and H12 are preserved for the two apo receptors. Specifically, the contact pairs H479-Y502 (H11–H12) and Y502-F506 (H12) of RORγ have a high contact strength and they are similar to Y326-L343 and L343-C347 in CAR. These three residues H479-Y502-F506 are collectively known as the HYF triplet, which forms a contact interaction network that is important for RORγ activity (Li et al., 2017; Ma et al., 2021). For example, inverse agonist may function by interacting with residue M358 and further disrupting contact interaction involving F506 (Marcotte et al., 2016). Previously, Y326-L343 in CAR was found to be critical to the agonist activity in the active conformation for CAR. In both CAR and RORγ, the His-Tyr lock stabilizes the position of helix H12 and contributes to the formation of the AF2 region. The disruption of H479-Y502 (H11–H12) through mutagenesis can prevent the coactivator from binding, thus reducing RORγ transcriptional activity (Kurebayashi et al., 2004). This is also supported by a high number of ligands forming contacts with both residues His479 and Tyr502 in the active conformation of RORγ in Figure 3A. In a previous study, the equivalent contact to His479-Tyr502 in CAR (Tyr326-Leu343) has been shown to be present in the apo conformation and strengthened by the binding of an agonist ligand (Pham et al., 2019a). Analogous to CAR, the His-Tyr lock is also present in our apo RORγ simulation with the average contact strength of 96.2%.

## Concluding Remarks

We studied the existing crystal structures of nuclear receptor RORγ, where various ligands (100+) interact with the binding pocket differently and result in an active or inactive conformation. By characterizing the protein conformation and protein-ligand interaction using residue contact interactions, we further performed a statistical analysis on these contact patterns. We identified the important residues at the binding pocket(s) that may be essential for interacting with potential inverse agonists. Besides studying the experimental data on a protein-ligand complex, we also used simulation to examine the apo structure ensemble and compared the high basal activity between RORγ and CAR. We found that the mechanism of constitutive activity is highly similar between them. These efforts lead to the understanding of the structure ensemble and protein-ligand interaction from a contact viewpoint, and they may facilitate future designs of inverse agonists for nuclear receptors.

## Data Availability

The original contributions presented in the study are included in the article/Supplementary Material, further inquiries can be directed to the corresponding author.
